# Clinical symptoms between severe and non-severe COVID-19 pneumonia: A protocol for systematic review and meta-analysis

**DOI:** 10.1097/MD.0000000000021618

**Published:** 2020-08-14

**Authors:** Peng Zheng, Lei Bao, Wei Yang, Jian-jiang Wang

**Affiliations:** a Department of intensive care unit; b Department of Critical Care Medicine, Jingjiang People's Hospital, Zhongzhou Road, Jingjiang, Taizhou City, Jiangsu Province, China.

**Keywords:** COVID-19, meta-analysis, non-severe, severe

## Abstract

**Background:**

Coronavirus disease 2019, (COVID-19) is a major problem in public health in the world. Up to June, 2020, the number of infections arising to 8,690,000 and cause 410,000 deaths all over the world. Identification the clinical symptoms from non-severe to severe is important for clinician. This meta-analysis aimed to compare the clinical symptoms between severe and non-severe COVID-19 pneumonia.

**Methods:**

Electronic databases including PubMed, EMBASE, Web of Science, China National Knowledge Infrastructure database, Wanfang Database and Chinese Biomedical Literature Database were searched from its inception to June 21, 2020. We only included severe versus non-severe COVID-19 pneumonia patients and pooled results were summarized by STATA 12.0 software.

Two researchers independently selected the study and assessed the quality of the included studies. The heterogeneity was measured by I^2^ tests (I^2^ < 50 indicates little heterogeneity, I^2^≥50 indicates high heterogeneity). Publication bias was ruled out by funnel plot and statistically assessed by Begg test (*P* > .05 as no publication bias).

**Results:**

Results will be published in relevant peer-reviewed journals.

**Conclusion:**

Our study aims to systematically present the clinical symptoms between non-severe and severe of COVID-19 patients, which will be provide clinical guidance for COVID-19 patients.

## 1 Introduction

Coronavirus disease 2019, (COVID-19) is a major problem in public health in the world.^[1,2]^ COVID-19 has spread throughout China and globally, as a pandemic.^[3,4]^ Up to June, 2020, the number of infections arising to 8,690,000 and cause 410,000 deaths all over the world.^[5]^ Clinical symptoms of COVID-19 mainly including fever, cough, and fatigue.^[6]^ Disease severity of COVID-19 could be divided into: mild, moderate, severe, critical, and death.^[7]^ Hinder the severity from mild or moderate to severe is top priority for clinicians.^[8]^ In a single-center case series, 26% of patients required admission to the intensive care unit (ICU) and 4.3% died.^[9]^ Thus, identifying the differences clinical symptoms related to severe versus non-severe COVID-19 is important for decision makers.

In this study, we systematically reviewed relevant published articles about severe and non-severe COVID-19 patients and used meta-analysis methods to analyses the difference clinical symptoms between severe versus non-severe COVID-19.

## 2 Methods and analysis

### 2.1 Study registration

We tried to plan, perform and report this meta-analysis in comply with Preferred Reporting Items for Systematic Review and Meta-analyses guideline, and registered in the Registry of Systematic Review/Meta-Analysis (https://www.researchregistry.com/browse-the-registry#registryofsystematicreviewsmeta-analyses/, No. reviewregistry937). And this study protocol was funded through a protocol registry. This study receives ethics approval from Jing-jiang People's Hospital.

### 2.2 Inclusion and exclusion criteria

In this study, both randomized controlled studies and cohort studies were included. The diagnosis of COVID-19 was confirmed as positive result for nasopharyngeal swab and respiratory pathogen nucleic acid test with high-throughput sequencing or real-time reverse transcriptase polymerase chain reaction (RT-PCR). Diagnostic criteria for COVID-19 severity is based on the CDCP (China) Diagnosis and Treatment of COVID-19. Mild and moderate were pooled into a non-severe group. Exclusion criteria were as follows:

(1) without insufficient data to pool;
(2) case reports;
(3) without gold standard for diagnosis of COVID-19.

### 2.3 Study search

Electronic databases including PubMed, EMBASE, Web of Science, China National Knowledge Infrastructure (CNKI) database, Wanfang Database and Chinese Biomedical Literature Database (CBM) were searched by 2 reviewers from its inception to June 21, 2020. Search terms included (Mesh “COVID-19” and key words “Novel coronavirus,” “Novel coronavirus 2019,” “2019 nCoV,” “COVID-19,” “Wuhan coronavirus”), and (Mesh “COVID-19” and key words “SARS-CoV-2,” “Wuhan pneumonia”). A manual search of the references of all the retrieved publications was conducted to identify additional studies. The uniformity between the 2 reviewers was tested using the kappa consistency: fair, 0.40 to 0.59; good, 0.60 to 0.74; and excellent, 0.75 or more.^[10]^

### 2.4 Study selection

**Figure 1:**
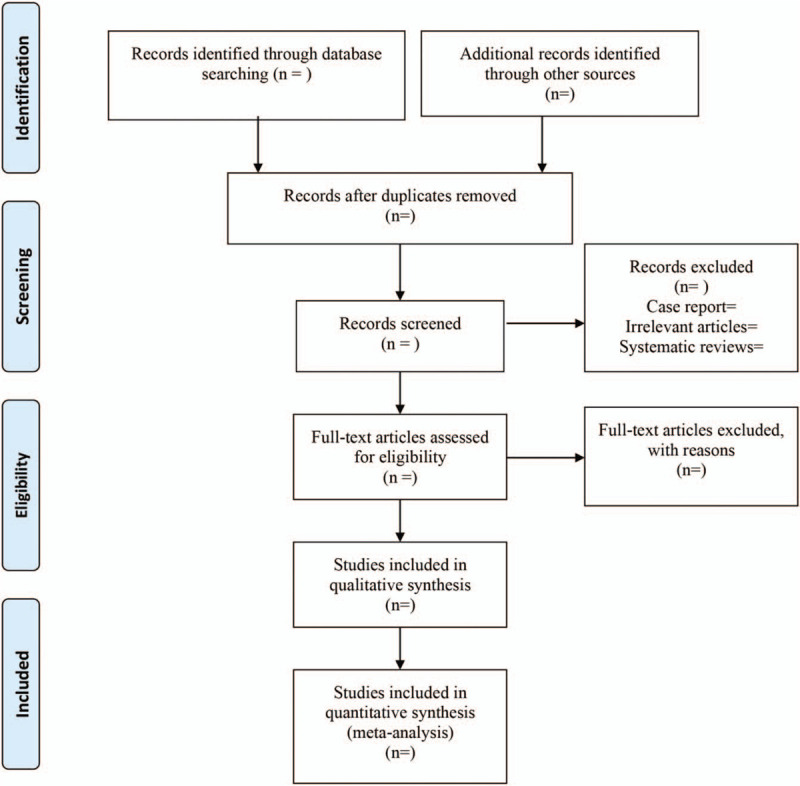
The flow diagram of procedure to select studies.

EndNote X9 (Thomson Reuters, Toronto, Ontario, Canada) was used for literature managing and records literature selection. Study selection was conducted independently by 2 reviewers (PZ and LB) and discrepant results were resolved by discussion until a unanimous decision was reached. The study flow chart is presented in Figure 1.

### 2.5 Data extraction

The following information was extracted: the first author, year of publication, number of the patients, mean age of patients, definition of severe and non-severe, onset time, contact history, and clinical symptoms (fever, cough, sore throat, tachycarida, rhinorrhea, nasal congestion, tachypnea, diarrhea, vomiting, myalgia or fatigue, hypoxemia, and chest pain), clinical laboratory outcomes (white blood cells, C-reactive protein (CRP), liver function, and renal function). Incidence between severe and non-severe patients were collected and recorded in Microsoft Excel (Microsoft Corp., Redmond, WA).

### 2.6 Risk of bias assessment

Two researchers (WY, BS, and J-jW) independently assessed the quality of the included trials based on Newcastle-Ottawa quality assessment scale (NOS) assessment tool.^[11]^ This tool mainly including 3 items: selection, comparability, and exposure. A “☆” rating system was used, and scores were ranged from 0 to 9. Studies with a score ≥7 were considered to be of high quality.

### 2.7 Data analysis

Stata 12.0 software (Stata Corp LLC, College Station, TX) was used for meta-analysis. For discontinuous variables, odds ratio (OR) was used to assess the effect of severe versus non-severe COVID-19. All results were presented as forest plot. Heterogeneity was quantified using I^2^, with I^2^ values >50% representing moderate heterogeneity. To explore sources of heterogeneity, subgroup analysis was performed by age of patients (<60 vs ≥ 60). Publication bias was ruled out by funnel plot and statistically assessed by Begg test (*P* > .05 as no publication bias).

## 3 Discussion

The aim of this study was to summarize the clinical symptoms between severe and non-severe COVID-19 pneumonia to provide guidance on disease development. This study has some highlights. First, this is the first systematic review and meta-analysis about the clinical symptoms between severe and non-severe COVID-19 pneumonia. In addition, we systematically searched the both English and Chinese databases to comprehensively selected the published papers. These methods demonstrate the reliability of our study. Consistency between reviewers were identified by kappa value. Finally, identify the difference between severe and non-severe was critically important for clinician to predict accurately of the disease development.

## Acknowledgments

We would thank for the Registry of Systematic Review/Meta-Analysis platform for registry for this meta-analysis.

## Author contributions

**Data curation:** Peng Zheng, Lei Bao.

**Formal analysis:** Peng Zheng, Wei Yang.

**Funding acquisition:** Lei Bao.

**Writing – original draft:** Wei Yang.
